# Evaluation of online videos to engage viewers and support decision-making for COVID-19 vaccination: how narratives and race/ethnicity enhance viewer experiences

**DOI:** 10.3389/fpubh.2023.1192676

**Published:** 2023-08-21

**Authors:** Holly B. Schuh, Rajiv N. Rimal, Robert F. Breiman, Peter Z. Orton, Matthew Z. Dudley, Lee-Sien Kao, Rikki H. Sargent, Shaelyn Laurie, Leo F. Weakland, James V. Lavery, Walter A. Orenstein, Janesse Brewer, Amelia M. Jamison, Jana Shaw, Robina Josiah Willock, Deborah A. Gust, Daniel A. Salmon

**Affiliations:** ^1^Department of Epidemiology, Johns Hopkins Bloomberg School of Public Health, Baltimore, MD, United States; ^2^Department of Health, Behavior and Society, Johns Hopkins Bloomberg School of Public Health, Baltimore, MD, United States; ^3^Hubert Department of Global Health, Rollins School of Public Health, Emory University, Atlanta, GA, United States; ^4^Independent Researcher, Mebane, NC, United States; ^5^Institute for Vaccine Safety, Johns Hopkins Bloomberg School of Public Health, Baltimore, MD, United States; ^6^Department of International Health, Johns Hopkins Bloomberg School of Public Health, Baltimore, MD, United States; ^7^Ideas42, New York, NY, United States; ^8^RIWI Corp., Toronto, ON, Canada; ^9^Center for Global Health Innovation, Atlanta, GA, United States; ^10^Center for Ethics, Emory University, Atlanta, GA, United States; ^11^Department of Medicine, Emory University, School of Medicine, Atlanta, GA, United States; ^12^Division of Infectious Diseases, Department of Pediatrics, The State University of New York (SUNY) Upstate Medical University, Syracuse, NY, United States; ^13^Department of Community Health and Preventive Medicine, Morehouse School of Medicine, Atlanta, GA, United States; ^14^Department of Psychology, Education Division, Gwinnett Technical College, Lawrenceville, GA, United States

**Keywords:** vaccine decision-making, vaccine hesitancy, COVID-19, personal narrative, race/ethnic congruence, internet-based intervention

## Abstract

**Background:**

Vaccine hesitancy has hampered the control of COVID-19 and other vaccine-preventable diseases.

**Methods:**

We conducted a national internet-based, quasi-experimental study to evaluate COVID-19 vaccine informational videos. Participants received an informational animated video paired with the randomized assignment of (1) a credible source (differing race/ethnicity) and (2) sequencing of a personal narrative before or after the video addressing their primary vaccine concern. We examined viewing time and asked video evaluation questions to those who viewed the full video.

**Results:**

Among 14,235 participants, 2,422 (17.0%) viewed the full video. Those who viewed a personal story first (concern video second) were 10 times more likely to view the full video (*p* < 0.01). Respondent–provider race/ethnicity congruence was associated with increased odds of viewing the full video (aOR: 1.89, *p* < 0.01). Most viewers rated the informational video(s) to be helpful, easy to understand, trustworthy, and likely to impact others' vaccine decisions, with differences by demographics and also vaccine intentions and concerns.

**Conclusion:**

Using peer-delivered, personal narrative, and/or racially congruent credible sources to introduce and deliver vaccine safety information may improve the openness of vaccine message recipients to messages and engagement.

## Introduction

Vaccine hesitancy and its role in vaccine uptake and also the subsequent control of vaccine-preventable diseases have become a major focus of research and practice (1). Since the convening of WHO's Strategic Advisory Working Group (SAGE) on vaccine hesitancy (2012) and the group's published definition of vaccine hesitancy in 2015 (2), researchers and practitioners in the vaccine community have continued to propose frameworks for measuring (3) and testing strategies to address vaccine hesitancy (3–7). In light of rising attention and efforts to address vaccine hesitancy over the past decade, vaccine hesitancy was formally recognized—prior to the start of the COVID-19 pandemic—as one of the top 10 threats to global health and security (8, 9).

The acceptance or refusal of COVID-19 vaccination—despite the widespread availability of vaccines—has been hampered by beliefs that COVID-19 does not present a serious health risk and a variety of concerns related to vaccine effectiveness and safety (10, 11). Along with sociodemographic factors (e.g., sex, education, race/ethnicity, and age), political affiliation, trust in public health authorities, and receiving the influenza vaccine in the prior year have been identified factors of COVID-19 vaccination (10–13). Despite a plethora of publications on vaccine hesitancy and the piqued interest of both experts and the public, to the best of our knowledge, there are few examples of rigorously tested vaccine communication strategies and interventions that have increased vaccine acceptance (14–16).

Experts agree that addressing vaccine hesitancy is context-specific, requiring tailored interventions that include a range of vaccine communication strategies (7). Patient–provider race/ethnicity concordance—defined as the occurrence of matching patient race/ethnicity and provider race/ethnicity—has been associated with an increased likelihood of care-seeking and continued care-seeking behaviors (17), as well as better patient–provider communication (18). Moreover, in science communication, listeners have been found to delay or not develop counter-arguments when listening to peer/personal narratives (19).

Our study was conducted as a part of the CDC-funded COVID-19 Vaccines Information Equity and Demand Creation (COVIED) program (20–22), a body of work designed to increase COVID-19 vaccination through the use of evidence-based, context-specific/tailored messaging. Based on our previous study on tailored vaccine education using racial/ethnic and gender diverse clinicians as credible sources and animation as a vehicle for conveying vaccine information (23), we conducted an internet-based, quasi-experimental study to evaluate the performance of 11 animated informational vaccine animation videos to address common vaccine attitudes and beliefs. We aimed to (1) evaluate the effect of using peer/personal narrative introductions (24) and (2) examine the role of race/ethnic congruence between the survey participant and a credible source (25) on the viewer engagement and their subsequent evaluation of the animated vaccine video intervention, randomizing on both video characteristics. We hypothesized that (1) introducing COVID-19 vaccine information with a personal narrative and (2) race/ethnic congruence between the survey participant and a credible source would be associated with an improvement in survey participant engagement, including an increase in the time spent viewing video content and positive ratings of video content. Additionally, we explored whether a credible source or participant's race/ethnicity was independently associated with viewer engagement and their evaluation of the intervention.

## Methods

### Study design

Using real-time interactive worldwide intelligence's (RIWI) patented Random Domain Intercept Technology (RDIT) (20–22), we implemented a national-level quasi-experimental design to evaluate 11 animated vaccine information videos with three variations of each based on different credible sources (i.e., clinical providers differentiating by race/ethnicity) who introduced and concluded each of the videos. A personal story video—narrated by an average peer of survey respondents, i.e., not a clinical provider—was created to precede or succeed eight (of the 11) videos that provided information on a common vaccine concern.

### Development of intervention

We developed content tailored to each of the following relevant sub-populations of COVID-19 vaccine decision-makers: primary caregivers of children (i.e., <18 years of age) who have concerns about COVID-19 vaccines for their children, primary caregivers who do not have concerns about COVID-19 vaccines, non-caregivers who have concerns about COVID-19 vaccines for themselves, and non-caregivers who do not have concerns about COVID-19 vaccines. Formative research for our study was conducted using analyses of RIWI RDIT-derived data from two other national-level rapid response surveys designed to ascertain the public's COVID-19 vaccination attitudes and beliefs (21, 22), rapid formative ethnography to elicit insights from sub-populations on the root causes and other related influencing factors of reluctance (and the intention) to receive a SARS-CoV-2 vaccine, and continuous message development and testing. Based on a similar process described elsewhere (23), we collaborated with a scriptwriter to develop evidence-driven video content grounded in the insights gained for each sub-population during our formative phase. We developed 11 animated vaccine informational videos that were refined through an iterative process between the scriptwriter and scientists to ensure content appropriateness, messaging true to current scientific knowledge, and application of defined behavioral theories (23).

All videos included an introduction and concluding message by a clinical provider as well as an animated informational video. Introduction and concluding recordings were performed by three clinicians representing different racial and ethnic backgrounds (Black, White, and Hispanic). The use of a personal story to introduce (or conclude) eight informational videos on COVID-19 vaccine concerns was developed based on the theory of change (26) that by establishing empathy and credibility and briefly addressing specific concerns followed by conveying disease risk and vaccine effectiveness, attitudes toward COVID-19 vaccination would become more positive. The effect is hypothesized to be greater if the message is introduced by a strong and personalized recommendation from a clinical provider (27).

### Data collection

We implemented the quasi-experiment from 06 December 2021 to 01 January 2022. We collected participant sociodemographic characteristics and determined video intervention eligibility based on answers to five questions regarding (1) caregiver status, (2) COVID-19 vaccination status, (3) race and ethnicity, (4) presence of COVID-19 vaccination concerns, and (5) intention to get the COVID-19 vaccine among the unvaccinated respondents. Based on answers to these questions, participants were assigned to appropriate message pathways (Supplementary Figure 1). Supplementary Table 1 presents all 11 possible response-specific videos.

### Video assignment

Child caregivers were stratified by those with any COVID-19 vaccine concern and those without a concern. All caregivers without any concern were assigned to view a video discussing benefits for the child (Child Benefit video). Caregivers who had a concern about the COVID-19 vaccine and infertility were randomly (3:1) assigned to view a concern video addressing their concern about infertility or the child benefit video. The remaining concerned caregivers were assigned to the child benefit video.

Among non-caregivers, any unvaccinated participant without COVID-19 vaccine concerns was assigned to a video discussing benefits for adults (adult benefit video). Vaccinated non-caregivers with a previous COVID-19 vaccine concern as well as any unvaccinated non-caregivers with a COVID-19 vaccine concern were asked a multiple-choice question—“what are/were your main concerns about the COVID-19 vaccine?”—and could select all that applied. Based on anticipated sample size limits, any participant indicating concern about vaccine ingredients (specifically, fetal cell lines) was automatically assigned to the concern video addressing this specific concern. Participants selecting only one concern were assigned to the associated concerned video. Participants who selected more than one concern (not about fetal cell lines) were randomly assigned to a concern video addressing one of the selected concerns. Participants who selected “other” concerns were assigned to view the adult benefit video. In order to test our theory of change using personal narrative to introduce vaccine risk and safety messages, one out of every eight participants assigned to view a concern video was randomly selected to view a personal story video before viewing the concern video (i.e., seven out of every eight viewed the personal story after).

Unvaccinated non-caregivers without concern about the COVID-19 vaccine were assigned to view the adult benefit video. Clinical provider race/ethnicity was randomly assigned (with equal probability) for all videos. Vaccinated non-caregivers without previous COVID-19 vaccine concerns were asked to participate in a brief survey about COVID-19 vaccine boosters.

### Outcomes of interest

We used a continuous measure of the length of time (in seconds) each respondent spent viewing their assigned video and a standardized measure of the proportion of the video viewed based on the total length of each video. The total length included the introduction, informational animation, and concluding message. We then created a dichotomous outcome variable identifying those who fully viewed their assigned video and those who did not. For respondents viewing a concern video, the total length of viewing time included both the concern video and the personal story video.

Participants who completed viewing their assigned video were asked to provide their level of agreement or disagreement using a five-point Likert scale (strongly agree, agree, neither agree nor disagree, disagree, and strongly disagree) with three statements to evaluate their assigned video:

(1) The video was helpful in making a vaccine decision.
(2) I trusted the information in the video.
(3) The video was easy to understand.

Viewers were asked to answer a fourth evaluation question (“how would this video influence others to get vaccinated?”) using a five-point Likert scale (much more likely, somewhat more likely, no impact, somewhat less likely, and much less likely). We opted for a neutral phrasing of the question to avoid any appearance of favor toward or against vaccination. Likert scales were dichotomized for analysis by combining “strongly agree” and “agree” or “much more likely” and “somewhat more likely” to form agree vs. disagree and likely vs. unlikely categories, respectively.

### Exposures of interest

The main exposure of interest was a categorical variable classifying respondents according to their assigned video type. For those assigned to any of the concern videos, separate categories were used to indicate whether the personal story video or concern video was viewed first. Sociodemographic characteristics of respondent age, sex, race and ethnicity, and COVID-19 vaccination status (vaccinated vs. unvaccinated), as well as provider's race and ethnicity, were measured. We created a dichotomous variable to indicate racial/ethnic congruence between the provider and the respondent. Respondent location was categorized according to nine regions using Centers for Disease Control and Prevention (CDC) definitions (28).

### Statistical analysis

For descriptive analyses, we compared distributions using chi-square and Fisher's exact tests for proportions, Wilcoxon rank-sum for non-parametric data, and Student's *t*-test for normally distributed data. No covariates had missingness of data exceeding 5%. Analyses were performed using the two-sided significance level (0.05). All analyses were conducted using Stata 16.1 (StataCorp, College Station, TX).

We used multivariable logistic regression to model the log odds of each of our dichotomous outcomes of interest: (1) viewing the assigned video in its entirety vs. not, and the respondent agreed (or disagreed) the following of the video; (2) is easy to understand; (3) is helpful for making vaccination decisions; (4) information is trustworthy; and (5) will influence others to get vaccinated. Table 1 shows video and respondent characteristics included in a backward stepwise selection process using 0.2 as the level of significance. We reviewed both the statistical significance of a fixed term used to control for survey date and interaction terms between provider–respondent racial congruence and provider race (and secondly, respondent race), and the results of a likelihood ratio test comparing extended and nested models that included (and subsequently excluded) a survey data fixed term. We reviewed the residual plots as well as Hosmer–Lemeshow chi-square estimates to evaluate the model goodness of fit. We used a robust variance estimator to adjust for clustering on respondent location (region).

**Table 1:** Demographic, survey, and COVID-19 vaccination characteristics among non-caregivers, stratified by completely viewing the assigned video or not.

	**Concern video** + **Personal video**				**Adult benefit video**			
	**Total**	**Dropped off viewing**	**Completed viewing**	***p*** **-value**	**Total**	**Dropped off viewing**	**Completed viewing**	***p*** **-value**
	***N*** = **4,043**	***N*** = **3,505**	***N*** = **538**		***N*** = **4,116**	***N*** = **3,529**	***N*** = **587**	
**Age category (years)**				<0.01				<0.01
18–25	1,241 (30.7%)	1,116 (89.9%)	125 (10.1%)		1,514 (36.8%)	1,315 (86.9%)	199 (13.1%)	
26–35	630 (15.6%)	568 (90.2%)	62 (9.8%)		683 (16.6%)	602 (88.1%)	81 (11.9%)	
36–45	365 (9.0%)	321 (87.9%)	44 (12.1%)		443 (10.8%)	369 (83.3%)	74 (16.7%)	
46–55	467 (11.6%)	378 (80.9%)	89 (19.1%)		443 (10.8%)	371 (83.7%)	72 (16.3%)	
56–64	478 (11.8%)	378 (79.1%)	100 (20.9%)		343 (8.3%)	281 (81.9%)	62 (18.1%)	
65–74	417 (10.3%)	343 (82.3%)	74 (17.7%)		245 (6.0%)	199 (81.2%)	46 (18.8%)	
75+	445 (11.0%)	401 (90.1%)	44 (9.9%)		445 (10.8%)	392 (88.1%)	53 (11.9%)	
**Gender**				<0.01				0.05
Male	2,403 (59.4%)	2,124 (88.4%)	279 (11.6%)		2,690 (65.4%)	2,327 (86.5%)	363 (13.5%)	
Female	1,640 (40.6%)	1,381 (84.2%)	259 (15.8%)		1,426 (34.6%)	1,202 (84.3%)	224 (15.7%)	
**Race and ethnicity**				<0.01				<0.01
White	2,397 (59.3%)	2,053 (85.6%)	344 (14.4%)		2,112 (51.3%)	1,795 (85.0%)	317 (15.0%)	
Black	484 (12.0%)	427 (88.2%)	57 (11.8%)		642 (15.6%)	536 (83.5%)	106 (16.5%)	
Alaskan Native	28 (0.7%)	27 (96.4%)	1 (3.6%)		42 (1.0%)	37 (88.1%)	5 (11.9%)	
Asian	444 (11.0%)	411 (92.6%)	33 (7.4%)		468 (11.4%)	427 (91.2%)	41 (8.8%)	
Hispanic/Latinx	359 (8.9%)	305 (85.0%)	54 (15.0%)		400 (9.7%)	342 (85.5%)	58 (14.5%)	
Multiple	120 (3.0%)	95 (79.2%)	25 (20.8%)		149 (3.6%)	122 (81.9%)	27 (18.1%)	
American Indian	81 (2.0%)	72 (88.9%)	9 (11.1%)		73 (1.8%)	64 (87.7%)	9 (12.3%)	
Other	130 (3.2%)	115 (88.5%)	15 (11.5%)		230 (5.6%)	206 (89.6%)	24 (10.4%)	
Survey date (median, IQR)	19 Dec 2021 (12–26 Dec)	19 Dec 2021 (12–26 Dec)	19 Dec 2021 (12–26 Dec)	0.78	19 Dec 2021 (12–26 Dec)	19 Dec 2021 (12–26 Dec)	19 Dec 2021 (12–26 Dec)	0.66
**COVID-19 vaccine concern** ^*^								0.42
No	-	-	-		2,930 (71.2%)	2,504 (85.5%)	426 (14.5%)	
Yes	4,043 (100%)	3,505 (100%)	538 (100%)		1,186 (28.8%)	1,025 (86.4%)	161 (13.6%)	
**Viewing order: concern vs. personal video first** ^**^				<0.01				
Concern first	3,515 (86.9%)	3,070 (87.3%)	445 (12.7%)		-	-	-	
Personal first	528 (13.1%)	435 (82.4%)	93 (17.6%)		-	-	-	
**Vaccination status**				<0.01				0.39
Vaccinated	2,888 (71.4%)	2,571 (89.0%)	317 (11.0%)		868 (21.1%)	752 (86.6%)	116 (13.4%)	
Unvaccinated	1,155 (28.6%)	934 (80.9%)	221 (19.1%)		3,248 (78.9%)	2,777 (85.5%)	471 (14.5%)	
**Received COVID-19 booster (among vaccinated only)**				<0.01				<0.01
Yes	1,661 (57.5%)	1,516 (91.3%)	145 (8.7%)		517 (59.6%)	453 (87.6%)	64 (12.4%)	
No, but plan to	832 (28.8%)	719 (86.4%)	113 (13.6%)		223 (25.7%)	180 (80.7%)	43 (19.3%)	
No, do not plan to	395 (13.7%)	336 (85.1%)	59 (14.9%)		128 (14.7%)	119 (93.0%)	9 (7.0%)	
**Intention to get COVID-19 vaccine (among unvaccinated)**				<0.01				0.05
Will definitely as soon as can	214 (18.5%)	197 (92.1%)	17 (7.9%)		723 (22.3%)	633 (87.6%)	90 (12.4%)	
Will likely as soon as can	80 (6.9%)	65 (81.2%)	15 (18.8%)		307 (9.5%)	273 (88.9%)	34 (11.1%)	
Will likely but not right away	151 (13.1%)	125 (82.8%)	26 (17.2%)		475 (14.6%)	391 (82.3%)	84 (17.7%)	
Will likely not	238 (20.6%)	185 (77.7%)	53 (22.3%)		583 (17.9%)	496 (85.1%)	87 (14.9%)	
Will definitely not	472 (40.9%)	362 (76.7%)	110 (23.3%)		1,160 (35.7%)	984 (84.8%)	176 (15.2%)	

^*^All respondents for concern + personal video viewing answered “yes” to COVID-19 vaccine concern.

^**^Viewing order only applies to concern + personal video viewing where the total viewing time—regardless of viewing order—includes both concern and personal story video lengths added together.

## Results

### Study population characteristics—descriptive analysis

Among 117,750 individuals who initially reached for participating, 75,616 (64.2%) completed the first five qualifying questions required for video assignment and 14,235 (18.8%) started to view the assigned video, allowing us to evaluate the length of time spent viewing the assigned video (e.g., the proportion of viewers who completed viewing their assigned video). Among those who started, 2,422 (17.0%) completed viewing the full video and answered at least one video and content quality or potential video utility evaluation question.

Among respondents assigned to view a concern and personal story video (4,043 non-caregivers with a COVID-19 vaccine concern), distributions of age, sex, race and ethnicity, COVID-19 vaccination status, COVID-19 booster status among the vaccinated, and intention to get the COVID-19 vaccine among the unvaccinated differed significantly by video viewing completion status (Table 1, left; all *p*-values < 0.01). Specifically, we observed higher proportions of video completion compared to demographic counterparts among respondents aged 46–55 and 56–64 years of age (19 and 21%, respectively), women (15.8%), self-reporting race and ethnicity as Hispanic/Latinx or Multiple (15–21%), those who were COVID-19 unvaccinated (19%), those who were COVID-19 vaccinated who are planning to get a COVID-19 booster (14%), and those who were COVID-19 unvaccinated and hesitant (delaying or refusing vaccination) (22%).

Among adult benefit video viewers (4,116 non-caregivers with or without a COVID-19 vaccine concern), we observed statistically significant differences in the distributions of age, race and ethnicity, and COVID-19 booster status among the vaccinated by video viewing completion status (Table 1, right; all *p*-values < 0.01). Mainly, those who were 36–74 years of age (~17%), self-identified race and ethnicity as Multiple (18%), Black (17%), and White or Hispanic/Latinx (15%) and were vaccinated but had not yet received the first COVID-19 booster but planned to (19%) had higher proportions of viewing compared to their younger (12–13%), Asian (9%), Alaskan Native (12%), American Indian (12%), and COVID-19 vaccinated (and have received a booster) (12%) counterparts (Table 1, right).

In Table 2 (left), among caregivers assigned to view the infertility concern video (*n* = 324), mostly 5–11-year-old children (36%) completed viewing than those aged 0–4 (23%) or 12–17 (18%) years (*p* = 0.02). No other significantly different distributions were found by video viewing completion status. Distributions of age (*p* < 0.01), sex (*p* = 0.01), and race and ethnicity (*p* < 0.01) differed significantly by child benefit video viewing completion status among 5,752 respondents where greater proportions of 36–64-year-olds (~9%), female (8%), and White (9%), Black (7%), Multiple (7%), Hispanic (7%), or American Indian (7%) completed viewing the video compared to those younger and older than the middle aged (~5%), male (7%), and Asian, Alaskan Native, or American Indian (all 5% completed viewing; Table 2, right).

**Table 2:** Demographic, survey, and COVID-19 vaccination characteristics among caregivers, stratified by completely viewing the assigned video or not.

	**Infertility video**				**Child benefit video**			
	**Total**	**Dropped off viewing**	**Completed viewing**	***p*** **-value**	**Total**	**Dropped off viewing**	**Completed viewing**	***p*** **-value**
	***N*** = **324**	***N*** = **241**	***N*** = **83**		***N*** = **5,752**	***N*** = **5,328**	***N*** = **424**	
**Age category (years)**				0.14				<0.01
18–25	39 (12.0%)	33 (85%)	6 (15%)		994 (17.3%)	938 (94.4%)	56 (5.6%)	
26–35	76 (23.5%)	61 (80%)	15 (20%)		1,223 (21.3%)	1,132 (92.6%)	91 (7.4%)	
36–45	89 (27.5%)	59 (66%)	30 (34%)		1,392 (24.2%)	1,276 (91.7%)	116 (8.3%)	
46–55	42 (13.0%)	29 (69%)	13 (31%)		932 (16.2%)	848 (91.0%)	84 (9.0%)	
56–64	16 (4.9%)	14 (88%)	2 (12%)		360 (6.3%)	326 (90.6%)	34 (9.4%)	
65–74	7 (2.2%)	4 (57%)	3 (43%)		199 (3.5%)	190 (95.5%)	9 (4.5%)	
75+	55 (17.0%)	41 (75%)	14 (25%)		652 (11.3%)	618 (94.8%)	34 (5.2%)	
**Gender**				0.43				0.01
Male	133 (41.0%)	102 (76.7%)	31 (23.3%)		2,947 (51.2%)	2,755 (93.5%)	192 (6.5%)	
Female	191 (59.0%)	139 (72.8%)	52 (27.2%)		2,805 (48.8%)	2,573 (91.7%)	232 (8.3%)	
**Race and ethnicity**				0.19				<0.01
White	146 (45.1%)	114 (78.1%)	32 (21.9%)		2,453 (42.6%)	2,233 (91.0%)	220 (9.0%)	
Black	54 (16.7%)	38 (70.4%)	16 (29.6%)		920 (16.0%)	853 (92.7%)	67 (7.3%)	
Alaskan Native	16 (4.9%)	13 (81.2%)	3 (18.8%)		129 (2.2%)	123 (95.3%)	6 (4.7%)	
Asian	24 (7.4%)	21 (87.5%)	3 (12.5%)		765 (13.3%)	729 (95.3%)	36 (4.7%)	
Hispanic/Latinx	35 (10.8%)	26 (74.3%)	9 (25.7%)		757 (13.2%)	706 (93.3%)	51 (6.7%)	
Multiple	22 (6.8%)	13 (59.1%)	9 (40.9%)		239 (4.2%)	222 (92.9%)	17 (7.1%)	
American Indian	7 (2.2%)	4 (57.1%)	3 (42.9%)		154 (2.7%)	144 (93.5%)	10 (6.5%)	
Other	20 (6.2%)	12 (60.0%)	8 (40.0%)		334 (5.8%)	317 (94.9%)	17 (5.1%)	
Missing	-	-	-		1 (0.0%)	1 (100.0%)	0 (0.0%)	
**COVID-19 vaccine concern**				0.43				0.57
No	144 (44.4%)	104 (72.2%)	40 (27.8%)		3,657 (63.6%)	3,382 (92.5%)	275 (7.5%)	
Yes	180 (55.6%)	137 (76.1%)	43 (23.9%)		2,095 (36.4%)	1,946 (92.9%)	149 (7.1%)	
**Child age (years)**				0.02				0.10
0–4	144 (44.4%)	111 (77.1%)	33 (22.9%)		2,270 (39.5%)	2,109 (92.9%)	161 (7.1%)	
12–17	79 (24.4%)	65 (82.3%)	14 (17.7%)		1,876 (32.6%)	1,750 (93.3%)	126 (6.7%)	
5–11	101 (31.2%)	65 (64.4%)	36 (35.6%)		1,606 (27.9%)	1,469 (91.5%)	137 (8.5%)	
Survey date, median (IQR)	18 Dec 2021 (12–26 Dec)	19 Dec 2021 (13–26 Dec)	17 Dec 2021 (11–25 Dec)	0.28	19 Dec 2021 (12–26 Dec)	19 Dec 2021 (12–26 Dec)	20 Dec 2021 (12–28 Dec)	0.08
**Vaccination status**				0.99				0.74
Vaccinated	117 (36.1%)	87 (74.4%)	30 (25.6%)		3,961 (68.9%)	3,666 (92.6%)	295 (7.4%)	
Unvaccinated	207 (63.9%)	154 (74.4%)	53 (25.6%)		1,791 (31.1%)	1,662 (92.8%)	129 (7.2%)	
**Received COVID-19 booster (among vaccinated only)**				0.89				0.25
Yes	35 (29.9%)	25 (71.4%)	10 (28.6%)		1,908 (33.2%)	1,762 (92.3%)	146 (7.7%)	
No, but plan to	44 (37.6%)	33 (75.0%)	11 (25.0%)		1,526 (26.5%)	1,407 (92.2%)	119 (7.8%)	
No, do not plan to	38 (32.5%)	29 (76.3%)	9 (23.7%)		527 (9.2%)	497 (94.3%)	30 (5.7%)	
**Intention to get COVID-19 vaccine (among unvaccinated)**				0.05				0.09
Will definitely as soon as can	14 (6.8%)	10 (71.4%)	4 (28.6%)		274 (4.8%)	255 (93.1%)	19 (6.9%)	
Will likely as soon as can	7 (3.4%)	7 (100.0%)	0 (0.0%)		177 (3.1%)	173 (97.7%)	4 (2.3%)	
Will likely but not right away	22 (10.6%)	13 (59.1%)	9 (40.9%)		292 (5.1%)	266 (91.1%)	26 (8.9%)	
Will likely not	38 (18.4%)	24 (63.2%)	14 (36.8%)		298 (5.2%)	276 (92.6%)	22 (7.4%)	
Will definitely not	126 (60.9%)	100 (79.4%)	26 (20.6%)		750 (13.0%)	692 (92.3%)	58 (7.7%)	

### Evaluation of viewing completion

In Table 3, the odds of completing the view of the assigned video are adjusted for the assigned video, provider race and ethnicity, and respondent race and ethnicity, sex, age, and COVID-19 vaccination status (vaccinated vs. unvaccinated). When compared to those who viewed the assigned concern video first (personal story second), viewers of the personal story first (concern video second) were ~10 times (*p* < 0.01) more likely to watch the complete informational animation. Viewers of the personal story first (concern video second) were also ~4.5 times (*p* < 0.01) more likely to watch the complete informational animation compared to viewers of the infertility concern video, as well as 20 times (*p* < 0.01) and nine times (*p* < 0.01) more likely to watch than the viewers of the child benefit and adult benefit videos, respectively. Unadjusted estimates of viewing time measures are provided in Supplementary Tables 2, 3.

**Table 3:** Association between fully viewing the assigned video or positive opinions of the viewed informational video and video and viewer sociodemographic characteristics.

	**Odds of fully viewing the assigned video**			**Odds the video is easier to understand**			**Odds the video is helpful for making vaccinations decisions**			**Odds the video will influence others to get vaccinated**			**Odds the viewer trusted the information in the video**		
	***n*** = **13,889**			***n*** = **2,378**			***n*** = **2,384**			***n*** = **2,416**			***n*** = **2,380**		
	**aOR** ^*^	***p*** **-value**	**95% CI**	**aOR** ^*^	***p*** **-value**	**95% CI**	**aOR** ^*^	***p*** **-value**	**95% CI**	**aOR** ^*^	***p*** **-value**	**95% CI**	**aOR** ^*^	***p*** **-value**	**95% CI**
**Viewed video (assigned)**															
Personal story first (concern second)^a^	Ref			Ref			Ref			Ref			Ref		
Concern video first (personal story second)^a^	**0.10**	<0.01	(0.07, 0.14)	0.93	0.80	(0.52, 1.66)	0.93	0.74	(0.6, 1.43)	**0.64**	<0.01	(0.48, 0.87)	1.08	0.65	(0.79, 1.47)
Infertility concern^b^	**0.22**	<0.01	(0.16, 0.30)	0.73	0.27	(0.42, 1.27)	**0.53**	<0.01	(0.39, 0.70)	**0.33**	<0.01	(0.24, 0.44)	**0.41**	<0.01	(0.33, 0.51)
Child benefit^c^	**0.05**	<0.01	(0.04, 0.07)	0.80	0.44	(0.45, 1.42)	1.07	0.80	(0.64, 1.77)	**0.64**	0.02	(0.44, 0.93)	1.40	0.09	(0.95, 2.05)
Adult benefit^d^	**0.11**	<0.01	(0.09, 0.14)	0.65	0.73	(0.06, 7.30)	1.33	0.76	(0.21, 8.42)	1.63	0.53	(0.35, 7.56)	1.74	0.59	(0.24, 12.76)
**Provider race**															
White	Ref			Ref			Ref			Ref			Ref		
Black	**0.42**	<0.01	(0.40, 0.45)	0.96	0.68	(0.81, 1.14)	1.05	0.58	(0.88, 1.25)	0.85	0.18	(0.67, 1.08)	0.87	0.16	(0.71, 1.06)
Hispanic	**0.18**	<0.01	(0.15, 0.22)	0.98	0.89	(0.71, 1.35)	0.96	0.63	(0.81, 1.14)	0.88	0.21	(0.73, 1.07)	0.90	0.42	(0.70, 1.16)
**Respondent race and ethnicity**															
White	Ref			Ref			Ref			Ref			Ref		
Black	0.99	0.96	(0.76, 1.29)	1.09	0.65	(0.74, 1.61)	**1.62**	<0.01	(1.39, 1.88)	**1.39**	<0.01	(1.12, 1.72)	1.20	0.22	(0.90, 1.60)
American Indian, Alaskan Native, Other	**0.73**	<0.01	(0.60, 0.90)	**0.56**	0.03	(0.33, 0.94)	1.03	0.88	(0.67, 1.60)	**0.68**	0.04	(0.48, 0.98)	**0.56**	<0.01	(0.41, 0.76)
Asian	**0.60**	<0.01	(0.45, 0.79)	0.83	0.50	(0.48, 1.44)	**2.10**	<0.01	(1.28, 3.44)	1.22	0.10	(0.96, 1.54)	1.39	0.20	(0.84, 2.28)
Hispanic/Latinx	0.99	0.97	(0.75, 1.32)	1.35	0.20	(0.85, 2.14)	**1.66**	<0.01	(1.19, 2.33)	**1.54**	0.01	(1.10, 2.14)	**1.54**	0.01	(1.11, 2.13)
Multiple	1.30	0.06	(0.99, 1.71)	0.67	0.05	(0.45, 0.99)	1.06	0.82	(0.63, 1.80)	0.95	0.82	(0.59, 1.52)	1.02	0.87	(0.79, 1.31)
**Respondent sex**															
Male	Ref			Ref			Ref			Ref			Ref		
Female	**1.29**	<0.01	(1.17, 1.43)	**1.27**	0.02	(1.05, 1.55)	0.99	0.90	(0.80, 1.22)	**1.29**	<0.01	(1.11, 1.50)	**1.26**	0.03	(1.02, 1.55)
**Respondent age (years)**															
18–35	Ref			Ref			Ref			Ref			Ref		
36–55	**1.44**	<0.01	(1.22, 1.70)	1.21	0.07	(0.98, 1.5)	0.79	0.12	(0.59, 1.06)	0.95	0.67	(0.76, 1.19)	1.02	0.90	(0.78, 1.33)
46–55	**1.62**	<0.01	(1.30, 2.02)	**1.53**	<0.01	(1.14, 2.06)	0.88	0.29	(0.71, 1.11)	1.10	0.40	(0.88, 1.36)	0.98	0.89	(0.72, 1.33)
56–64	**1.76**	<0.01	(1.42, 2.19)	1.10	0.64	(0.73, 1.66)	0.78	0.12	(0.56, 1.07)	0.93	0.77	(0.58, 1.50)	0.78	0.12	(0.57, 1.07)
65–74	**1.58**	<0.01	(1.32, 1.90)	1.14	0.46	(0.80, 1.63)	0.90	0.47	(0.66, 1.21)	1.14	0.46	(0.80, 1.63)	0.84	0.34	(0.58, 1.20)
75+	0.95	0.73	(0.71, 1.26)	**0.47**	<0.01	(0.34, 0.64)	**0.70**	0.02	(0.52, 0.94)	**0.53**	<0.01	(0.38, 0.75)	**0.66**	0.01	(0.50, 0.89)
**COVID-19 vaccination status**															
Vaccinated	Ref			Ref			Ref			Ref			Ref		
Unvaccinated	**1.32**	0.01	(1.09, 1.60)	**0.31**	<0.01	(0.22, 0.44)	**0.30**	<0.01	(0.24, 0.37)	**0.26**	<0.01	(0.21, 0.34)	**0.16**	<0.01	(0.13, 0.21)
B0—intercept	**1.98**	<0.01	(1.51, 2.60)	**5.60**	<0.01	(3.38, 9.30)	1.53	0.19	(0.81, 2.89)	**2.54**	<0.01	(1.60, 4.02)	**1.84**	0.02	(1.12, 3.02)

Variance calculated using clustering term for geographic region (CDC definitions).

^*^Statistically significant point estimates (p < 0.05) are bolded.

^a^Concern videos include: Benefits of vaccination for pregnancy, COVID-19 is not that serious, Concerned about common side effects, Concerned about vaccine ingredients, Concerned about fetal cell line, Concerned about general safety (of COVID-19 vaccines), Vaccines were developed too fast, and Serious side effects (see Supplementary Table 1). The total viewing time—regardless of viewing order—includes both Concern and Personal Story video lengths added together.

^b^Video for those Concerned about infertility (Supplementary Table 1).

^c^Video about the Benefits of vaccination for children.

^d^Video about the Benefits of vaccination for adults.

Those viewing introduction/concluding material presented by the Black or Hispanic physician had lower odds of fully viewing the assigned video (58%, *p* < 0.01; 82%, *p* < 0.01) than those viewing videos presented by the White physician (Table 3). There was title difference between the adjusted and crude odds ra8os for respondent sex, age, race and ethnicity, and COVID-19 vaccina8on status except that viewers who self-iden8fied as American Indian (AI), Alaskan Na8ve (AN), or Other had lower odds of fully viewing the assigned video (27%, *p* < 0.01) compared to their White counterparts as did Asian viewers (40% lower, *p* < 0.01). Those reporting a multi-racial and/or ethnic identity had greater odds of completely viewing the assigned video (aOR 1.30, 95% CI 0.99, 1.71) though the relationship had borderline statistical significance (*p* = 0.06). Women aged 36–74 years and unvaccinated viewers had increased odds of fully viewing the assigned video compared to their men aged 18–35 years and vaccinated counterparts (aOR_female_: 1.29; aOR_36−74−years−old_ range: 1.44–1.76; aOR_Unvaccinated_: 1.32; all *p* < 0.01).

When adjusting for racial congruence between the provider and the viewer/respondent (Table 4), the odds of fully viewing the assigned video were lower for all videos compared to personal story video first (concern video second) viewers (all *p*-values < 0.01). Racial congruence was associated with increased odds of fully viewing a video (aOR: 1.89, *p* < 0.01).

**Table 4:** Association between fully viewing the assigned video or positive opinions of the viewed informational video and video and viewer sociodemographic characteristics that include provider-respondent racial congruence.

	**Odds of fully viewing the assigned video**			**Odds the video is easier to understand**			**Odds the video is helpful for making vaccinations decisions**			**Odds the video will influence others to get vaccinated**			**Odds the viewer trusted the information in the video**		
	***n*** = **13,890**			***n*** = **2,378**			***n*** = **2,384**			***n*** = **2,416**			***n*** = **2,380**		
	**aOR**	***p*** **-value**	**95% CI**	**aOR**	***p*** **-value**	**95% CI**	**aOR**	***p*** **-value**	**95% CI**	**aOR**	***p*** **-value**	**95% CI**	**aOR**	***p*** **-value**	**95% CI**
**Viewed video (assigned)**															
Personal story first (concern second)^a^	Ref			Ref			Ref			Ref			Ref		
Concern video first (personal story second)^a^	**0.11**	<0.01	(0.08, 0.15)	0.90	0.72	(0.51, 1.59)	0.95	0.81	(0.64, 1.43)	**0.65**	<0.01	(0.48, 0.87)	1.08	0.60	(0.8, 1.46)
Infertility concern^b^	**0.25**	<0.01	(0.19, 0.32)	0.66	0.08	(0.41, 1.04)	**0.57**	<0.01	(0.44, 0.74)	**0.33**	<0.01	(0.25, 0.44)	**0.40**	<0.01	(0.31, 0.53)
Child benefit^c^	**0.06**	<0.01	(0.05, 0.08)	0.78	0.39	(0.45, 1.36)	1.16	0.55	(0.71, 1.89)	**0.67**	0.04	(0.46, 0.97)	1.44	0.05	(0.99, 2.08)
Adult benefit^d^	**0.12**	<0.01	(0.1, 0.15)	0.67	0.74	(0.07, 6.77)	1.75	0.51	(0.34, 9.18)	1.87	0.37	(0.47, 7.36)	1.98	0.44	(0.35, 11.32)
**Provider and respondent races are congruent**															
No	Ref			Ref			Ref			Ref			Ref		
Yes	**1.89**	<0.01	(1.62, 2.20)	1.03	0.82	(0.79, 1.36)	1.00	1.00	(0.8, 1.25)	**1.14**	0.03	(1.01, 1.29)	1.06	0.46	(0.90, 1.25)
**Respondent sex**															
Male	Ref			Ref			Ref			Ref			Ref		
Female	**1.28**	<0.01	(1.15, 1.41)	**1.29**	0.01	(1.05, 1.58)	0.97	0.78	(0.79, 1.20)	**1.29**	<0.01	(1.11, 1.50)	**1.25**	0.03	(1.02, 1.54)
**Respondent age (years)**															
18–35	Ref			Ref			Ref			Ref			Ref		
36–55	**1.38**	<0.01	(1.21, 1.58)	1.20	0.10	(0.97, 1.48)	0.74	0.05	(0.55, 1)	0.92	0.48	(0.72, 1.16)	0.98	0.86	(0.76, 1.26)
46–55	**1.56**	<0.01	(1.3, 1.88)	**1.51**	<0.01	(1.14, 2.00)	0.80	0.06	(0.63, 1.01)	1.03	0.79	(0.82, 1.29)	0.93	0.63	(0.69, 1.25)
56–64	**1.73**	<0.01	(1.45, 2.07)	1.11	0.62	(0.73, 1.69)	0.70	0.05	(0.49, 1.00)	0.89	0.63	(0.54, 1.45)	0.73	0.08	(0.51, 1.04)
65–74	**1.53**	<0.01	(1.35, 1.74)	1.15	0.44	(0.81, 1.62)	0.79	0.13	(0.58, 1.07)	1.06	0.74	(0.75, 1.51)	0.77	0.16	(0.54, 1.11)
75+	0.94	0.69	(0.71, 1.26)	**0.42**	<0.01	(0.31, 0.55)	**0.62**	<0.01	(0.46, 0.83)	**0.47**	<0.01	(0.33, 0.65)	**0.57**	<0.01	(0.44, 0.73)
**COVID-19 vaccination status**															
Vaccinated	Ref			Ref			Ref			Ref			Ref		
Unvaccinated	**1.33**	<0.01	(1.12, 1.59)	**0.31**	<0.01	(0.22, 0.44)	**0.29**	<0.01	(0.24, 0.35)	**0.26**	<0.01	(0.2, 0.33)	**0.16**	<0.01	(0.12, 0.20)
B0—intercept	**0.70**	<0.01	(0.56, 0.88)	**5.43**	<0.01	(2.98, 9.89)	**1.89**	0.04	(1.03, 3.48)	**2.45**	<0.01	(1.57, 3.82)	**1.83**	0.02	(1.12, 2.99)

^a^Concern videos include: Benefits of vaccination for pregnancy, COVID-19 is not that serious, Concerned about common side effects, Concerned about vaccine ingredients, Concerned about fetal cell line, Concerned about general safety (of COVID-19 vaccines), Vaccines were developed too fast, and Serious side effects (see Supplementary Table 1). The total viewing time—regardless of viewing order—includes both Concern and Personal Story video lengths added together.

^b^Video for those Concerned about infertility (Supplementary Table 1).

^c^Video about the Benefits of vaccination for children.

^d^Video about the Benefits of vaccination for adults. The bold values are used where the associated *p*-value indicates statistical significance <0.05.

### Evaluation of video content (post-viewing)

Overall, the odds of evaluating the video positively (easier to understand, helpful for making vaccination decisions, providing trusted information, and influencing others to get vaccinated) were greater among those who viewed the personal story first (concern video second) though the adjusted odds ratio was statistically significant only when asking about the video's influence on others to get vaccinated (1.6 times greater odds, *p* < 0.01; Table 3). Independently, the provider's race was not significantly associated with a positive evaluation of the videos, whereas respondent race/ethnicity was significantly associated with positive evaluation, and mainly Black, Asian, and Hispanic/LatinX have greater odds (aOR: 1.4–2.1 where *p* < 0.01) of evaluating the videos positively [and American Indian or Alaskan Native (AI/AN)] or others have a lower odds (≤46% lower odds where *p* < 0.01) than their White counterparts (Table 3). Racial congruence was associated with increased odds of evaluating the video as influencing others to get vaccinated (Table 4; aOR: 1.14, *p* = 0.03). When adjusting for racial congruence, female viewers had greater odds (1.3 greater odds compared to males, all *p* ≤ 0.03) of positively evaluating their assigned videos whereas older adults (75+ years of age; ≤53% lower odds where *p* ≤ 0.02) and the unvaccinated ones had lower odds (≤84% lower odds where *p* < 0.01; Table 4).

### Sub-analyses—potential confounding and effect modifying effects

There was no evidence of a confounding effect of survey time on the primary relationship of interest (*p* = 0.97). Moreover, we found no evidence that provider race (or respondent race) acts as an effect modifier on the relationship between provider–respondent racial congruence and our outcomes of interest (*p* > 0.40 and *p* > 0.30 for provider and respondent race, respectively).

## Discussion

Using peer-delivered, personal narratives to introduce vaccine safety information may increase the likelihood that viewers will engage with informational vaccine videos. Despite fewer clear benefits in the likelihood of videos receiving positive evaluations, personal story videos had a consistently improved effect on the likelihood that viewers thought their respective videos would influence others to get vaccinated. Emotional engagement—an important part of communication strategies developed to engage the public for fostering vaccine confidence—has been a central part of health behavior change research and practice (23, 29, 30). Emotional engagement and transparent communication likely serve as important tools for messaging and vaccination program administration, particularly during periods of heightened collective and diverse emotions among the public such as the COVID-19 pandemic (30). Moreover, the use of personal narratives may foster learning environments of openness (open-mindedness) (31) for vaccine messages designed to build general confidence and understanding of evidence-based medicine (32). In light of these findings and previous research that has highlighted the polarization of social media content between positive and negative-toned content on vaccines, especially vaccine-hesitant topics (33), our findings on the influence of peer-delivered, personal narrative to introduce vaccine safety information may be an important area of further research.

Our findings suggest the importance of racial congruence between the patient and the provider in vaccine safety communication and also further support the importance of identifying sub-population attitudes (e.g., by race and ethnicity) and tailoring messages. Race and ethnicity have been identified as factors associated with COVID-19 (and other) vaccine uptake and vaccine hesitancy (25, 34–36). In general, healthcare providers who communicate effectively with patients are known influencers of vaccine uptake (25, 37, 38). However, some evidence supports that persons self-identifying as non-Hispanic White are more likely to receive a healthcare provider's recommendation than racial and ethnic minorities (37). Studies have shown that patients who are racially and/or ethnically concordant with their provider report greater satisfaction, levels of trust, and perceived quality of care (17). Although concordance has been found to affect patients' clinical encounter experiences and relates to better patient–physician communication, there is no general consensus on the positive effect of racial/ethnic patient–provider concordance on patient outcomes (nor specifically on effective vaccine communication) (18). Our study indicates support for this theory.

We found that while unvaccinated respondents were more likely to fully view their assigned video compared to vaccinated respondents, they were also less likely to give positive feedback on video content and usefulness. The majority of unvaccinated respondents (62%) agreed or strongly agreed that the video content was easy to understand, but greater proportions of unvaccinated respondents disagreed or strongly disagreed that the videos were helpful for making vaccine decisions or influencing others to get vaccinated or that the information was trusted than agreed or strongly agreed (69, 68, and 74%, respectively). Even before the COVID-19 pandemic, vaccine hesitancy and refusal were identified as public health concerns (4, 8), with emotion-driven vaccine beliefs spreading across (and even flourish) during the pandemic (30, 39). Regardless of their interest to view the content of our informational videos, deliberate efforts to engage viewers in a positive manner may rather activate emotions that decrease the likelihood of positive feedback (30). Resistance to vaccination is complex, and positive vaccine messages may have unintended and undesirable consequences (25, 40). Rigorous approaches to both measuring latent vaccination attitudes and beliefs and testing interventions for their effect on vaccination behavior (i.e., uptake) are needed and must take the psychology behind health decision-making into consideration (23, 25, 41, 42).

### Limitations

Selection bias may have been introduced due to the opt-in, unincentivized design of the study. We explored the distribution of sociodemographic characteristics (Supplementary Table 4) among those who were lost to follow-up (i.e., early drop-out and did not get assigned to a video or did not start viewing the assigned video), noting that the proportion of these individuals are more vaccinated (77.3 vs. 55.0%, *p* < 0.01) and have fewer reported COVID-19 vaccine concerns than those described in our study sample (26.3 vs. 52.7%, *p* < 0.01). This may strengthen our findings that suggest the usefulness of our vaccine communication videos among specific sub-populations of the American public, noting that the distribution of intentions to complete the COVID-19 vaccine series or booster were similar between those lost to follow-up and those in our study (Supplementary Table 4). We note that our sample size may have limited our ability to identify statistically significant differences in the odds of viewing the entire assigned video among the multi-racial and/or ethnic subgroup compared to their racial/ethnic counterparts. The focus of this study was on the evaluation of the whole package of information and associations with demographic characteristics and vaccine intentions and concerns. Thus, we were not able to determine if vaccination rates improved in persons who viewed full videos compared to others. Further research is needed to evaluate this question.

## Public health implications

Our study findings further support the importance of tailoring vaccine communication strategies to sub-population vaccine attitudes by delivering vaccine messages through trusted, race/ethnicity-congruent providers or other trusted health authorities. Introducing vaccine safety information with peer-delivered, personal narratives may improve the openness of vaccine message recipients to vaccine messages and engagement. Further research is needed to evaluate the effect of vaccine safety informational video packages on vaccine uptake. Additionally, further formative work is needed to explore message engagement among sub-populations that maintain fewer positive views of vaccine safety information.

## Data availability statement

The raw data supporting the conclusions of this article will be made available by the authors, without undue reservation.

## Ethics statement

This study was reviewed by the Institutional Review Board at John Hopkins Bloomberg School of Public Health. Written informed consent and approval was not required as the study was determined to not be human subject research.

## Author contributions

HS, RR, RB, PO, MD, L-SK, RS, SL, LW, JL, WO, JB, AJ, JS, RJ, DG, and DS contributed to the conception and/or the design of the work represented in this paper. RS and SL were a part of the acquisition of data. HS, RB, MD, RS, LW, WO, and DS contributed to the analysis and interpretation of data. HS and DS drafted the work. All authors revised the work critically and gave final approval of the version to be published.
