# Supplementary material for: Evaluation of online videos to engage viewers and support decision-making for COVID-19 vaccination: how narratives and race/ethnicity enhance viewer experiences
Source: Front Public Health. 2023 Aug 21;11:1192676. doi: 10.3389/fpubh.2023.1192676 (PMC10475941; doi:10.3389/fpubh.2023.1192676)
Supplement: Supplementary file 1 [file Data_Sheet_1.pdf]

Supplemental Figure 1. Video intervention assignment flowchart

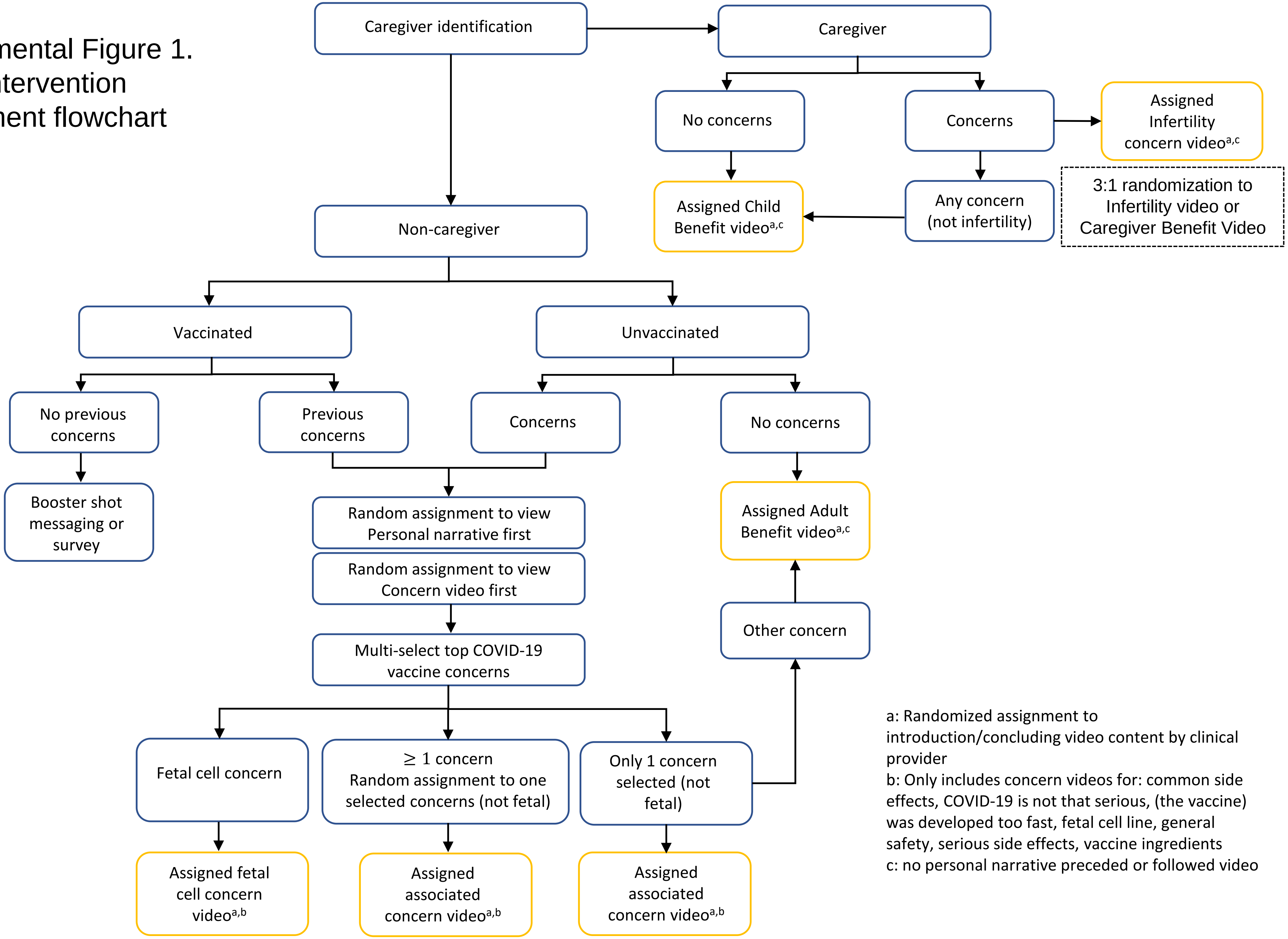

a: Randomized assignment to introduction/concluding video content by clinical provider
b: Only includes concern videos for: common side effects, COVID-19 is not that serious, (the vaccine) was developed too fast, fetal cell line, general safety, serious side effects, vaccine ingredients
c: no personal narrative preceded or followed video

Supplemental Figure 2.
Study flowchart

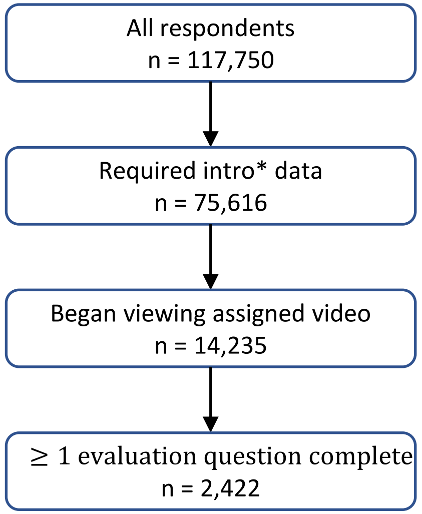

* Required introductory question on: COVID-19 vaccination status; race and ethnicity; COVID-19 vaccine concern

Supplemental table 1. Type of viewer/respondent and video assignment

Video name	COVID-19 vaccine concern(s) (yes/no)	Full video length (in seconds)
Benefits of vaccination for adults (adult benefit)	No	132
Benefits of vaccination for children[a] (child benefit)	No	181
Concerned about infertility[a]	Yes	216
Benefits of vaccination for pregnancy[b]	Yes	150
COVID-19 is not that serious[b]	Yes	246
Concerned about common side effects[b]	Yes	233
Concerned about vaccine ingredients[b]	Yes	279
Concerned about fetal cell line[b]	Yes	232
Concerned about general safety[b]	Yes	247
Vaccines developed to fast[b]	Yes	296
Serious side effects[b]	Yes	391

a: indicates the respondent is the primary caregiver of a child 0-17 years of age

b: a concern video randomly assigned to preceding or proceeding a personal narrative story; full video lengths include length of personal narrative (about 108 seconds of viewing time)

Supplemental table 2. Viewing time and proportion of viewers who dropped off while viewing the assigned video, stratified by five general video viewing assignment flows

	Total	Non-caregivers			Caregivers		p-value
		Personal first*,a	Concern first*,a	Adult benefit[b]	Infertility[b]	Child benefit[b]	
	N=14,235	N=528	N=3,515	N=4,116	N=324	N=5,752	
Viewing time (seconds), median (iqr)	14 (5-122)	24 (6-114)	15 (5-133)	12 (4-85.5)	23 (6-199)	15 (5-129)	<0.01
Percent of video viewed, median (iqr)	8.3 (2.8-59.5)	21.2 (5.3-71.25)	5.58 (1.72-53.85)	9.1 (3.3-67.05)	11.1 (3-100)	8.5 (2.8-72.3)	<0.01
Dropped off while viewing assigned video							<0.01
No	1,632 (11.5%)	93 (17.6%)	445 (12.7%)	587 (14.3%)	83 (25.6%)	424 (7.4%)	
Yes	12,603 (88.5%)	435 (82.4%)	3,070 (87.3%)	3,529 (85.7%)	241 (74.4%)	5,328 (92.6%)	

* Total viewing time is calculated by adding seconds of Personal and Concern video viewed

a: Only includes concern videos for: common side effects, COVID-19 is not that serious, (the vaccine) was developed too fast, fetal cell line, general safety, serious side effects, vaccine ingredients

b: no personal narrative preceded or followed video

Supplemental table 3. Viewing time and proportion of viewers who dropped off while viewing the assigned video, stratified by specific assigned Concern video

	Total N=4,007	Common side effects N=448	COVID-19 is not serious N=257	Developed too fast N=538	Fetal cells N=585	General safety N=739	Serious side effects N=651	Vaccine ingredients N=465	Infertility[a] N=324	p-value
Viewing time (seconds), median (iqr)	18 (5-139)	11 (5-127)	10 (4-39)	18.5 (5-186)	22 (5-121)	25 (6-140)	18 (5-240)	14 (5-171)	23 (6-199)	<0.01
Percent of video viewed	6.48 (2.02-55.87)	4.72 (2.15-54.51)	4.07 (1.63-15.85)	6.25 (1.69-62.84)	9.48 (2.16-52.16)	10.12 (2.43-56.68)	4.6 (1.28-61.38)	5.38 (1.79-61.29)	11.1 (3-100)	<0.01
Dropped off while viewing assigned video										<0.01
No	621 (15.5%)	70 (15.6%)	21 (8.2%)	70 (13.0%)	98 (16.8%)	139 (18.8%)	76 (11.7%)	64 (13.8%)	83 (25.6%)	
Yes	3,386 (84.5%)	378 (84.4%)	236 (91.8%)	468 (87.0%)	487 (83.2%)	600 (81.2%)	575 (88.3%)	401 (86.2%)	241 (74.4%)	

a: no personal narrative preceded or followed video

Supplemental table 4. Demographic, survey, and COVID-19 vaccination characteristics among **respondents who did not start viewing** their assigned video

	Started viewing assigned video			
	Total<br>N=75,579	Yes<br>N=14,235	No<br>N=61,344	p-value
Age category (years)				<0.01
18-25	21,372 (28.3%)	3,788 (26.6%)	17,584 (28.7%)	
26-35	14,420 (19.1%)	2,612 (18.3%)	11,808 (19.2%)	
36-45	10,630 (14.1%)	2,289 (16.1%)	8,341 (13.6%)	
46-55	8,443 (11.2%)	1,884 (13.2%)	6,559 (10.7%)	
56-64	6,576 (8.7%)	1,197 (8.4%)	5,379 (8.8%)	
65-74	5,958 (7.9%)	868 (6.1%)	5,090 (8.3%)	
75+	8,180 (10.8%)	1,597 (11.2%)	6,583 (10.7%)	
Gender				<0.01
Male	44,971 (59.5%)	8,173 (57.4%)	36,798 (60.0%)	
Female	30,608 (40.5%)	6,062 (42.6%)	24,546 (40.0%)	
Race and ethnicity				<0.01
White	40,397 (53.5%)	7,108 (49.9%)	33,289 (54.3%)	
Black	9,861 (13.0%)	2,100 (14.8%)	7,761 (12.7%)	
Alaskan Native	1,021 (1.4%)	215 (1.5%)	806 (1.3%)	
Asian	9,329 (12.3%)	1,701 (11.9%)	7,628 (12.4%)	
Hispanic / Latinx	7,716 (10.2%)	1,551 (10.9%)	6,165 (10.0%)	
Multiple	2,403 (3.2%)	530 (3.7%)	1,873 (3.1%)	
American Indian	1,319 (1.7%)	315 (2.2%)	1,004 (1.6%)	
Other	3,532 (4.7%)	714 (5.0%)	2,818 (4.6%)	
Missing	1 (0.0%)	1 (0.0%)	0 (0.0%)	
COVID-19 vaccine concern				<0.01
No	51,964 (68.8%)	6,731 (47.3%)	45,233 (73.7%)	
Yes	23,614 (31.2%)	7,504 (52.7%)	16,110 (26.3%)	
Missing	1 (0.0%)	0 (0.0%)	1 (0.0%)	
Survey date	19 Dec 2021 (12-26 Dec)	19 Dec 2021 (12-26 Dec)	19 Dec 2021 (12-26 Dec)	0.55
Vaccination status				<0.01
Vaccinated	55,283 (73.1%)	7,834 (55.0%)	47,449 (77.3%)	
Unvaccinated	20,296 (26.9%)	6,401 (45.0%)	13,895 (22.7%)	
Received COVID-19 booster (among vaccinated only)				<0.01
Yes	30,634 (55.4%)	4,121 (52.6%)	26,513 (55.9%)	
No, but plan to	18,432 (33.3%)	2,625 (33.5%)	15,807 (33.3%)	
No, do not plan to	6,217 (11.2%)	1,088 (13.9%)	5,129 (10.8%)	
Intention to get COVID-19 vaccine (among unvaccinated)				<0.01
Will definitely as soon as can	4,167 (20.5%)	1,225 (19.1%)	2,942 (21.2%)	
Will likely as soon as can	1,958 (9.6%)	571 (8.9%)	1,387 (10.0%)	
Will likely but not right away	2,968 (14.6%)	940 (14.7%)	2,028 (14.6%)	
Will likely not	3,389 (16.7%)	1,157 (18.1%)	2,232 (16.1%)	
Will definitely not	7,814 (38.5%)	2,508 (39.2%)	5,306 (38.2%)	
